# The Role of Cholesterol in Chronic Lymphocytic Leukemia Development and Pathogenesis

**DOI:** 10.3390/metabo13070799

**Published:** 2023-06-27

**Authors:** Alana M. White, Oliver G. Best, Anya K. Hotinski, Bryone J. Kuss, Lauren A. Thurgood

**Affiliations:** Molecular Medicine and Genetics, College of Medicine and Public Health, Flinders University, Bedford Park, SA 5042, Australiagiles.best@flinders.edu.au (O.G.B.);

**Keywords:** chronic lymphocytic leukemia (CLL), statins, cancer, dyslipidemia, LDL, HDL

## Abstract

Cholesterol has many critical functions in cells. It is a key component of membranes and cell-signalling processes, and it functions as a chemical precursor in several biochemical pathways, such as Vitamin D and steroid synthesis. Cholesterol has also been implicated in the development and progression of various cancers, in which it is thought to promote cell proliferation, migration, and invasion. Chronic lymphocytic leukemia (CLL) is an example of a lipid-avid cancer that relies on lipid metabolism, rather than glycolysis, to fuel cell proliferation. However, data regarding the role of cholesterol in CLL are conflicting. Studies have shown that dyslipidaemia is more common among CLL patients than age-matched healthy controls, and that CLL patients who take cholesterol-lowering drugs, such as statins, appear to have improved survival rates. Therefore, defining the roles of cholesterol in CLL may highlight the importance of monitoring and managing hyperlipidaemia as part of the routine management of patients with CLL. In this review, we discuss the roles of cholesterol in the context of CLL by examining the literature concerning the trafficking, uptake, endogenous synthesis, and intracellular handling of this lipid. Data from clinical trials investigating various classes of cholesterol and lipid-lowering drugs in CLL are also discussed.

## 1. Introduction—The Role of Cholesterol in Cancer

For almost a century, it has been acknowledged that nutrients play an important role in cancer [1]. There is growing evidence that cholesterol, a type of lipid, plays a significant role in carcinogenesis. Numerous studies, both in vitro and clinical, have found that hypercholesterolemia can affect the development of cancer [2]. For example, a 10mg/dL increase in cholesterol levels was associated with a 9% increase in prostate cancer recurrence [3]. In a study of almost 300,000 Danish patients, lowering cholesterol levels through the administration of drugs such as statins was found to cause a slight reduction in cancer-related mortality of patients with 13 different types of cancer [4].

There is evidence that serum cholesterol levels start to decline up to 6 years before the detection of colon cancer and chronic myeloid leukemia [5,6], suggesting that cholesterol levels may be an early predictor of cancer development for some tumours. Conversely, epidemiological studies have shown that increased serum cholesterol is positively correlated with a higher incidence of some cancers, such as those of the colon, prostate, and testes [2,7]. While data from these studies suggest that cholesterol levels may be critical in some tumours, a review by Ding et al. [8] suggests that the link between cancer progression and cholesterol is much more complicated than a simple two-factor association, and that other factors, such as the cholesterol requirement for the tissue of cancer origin and dietary habits, may be more critical. For example, in patients with bladder cancer, more aggressive disease was observed among patients taking statins [9], and large studies carried out in the 1980s determined that low cholesterol levels were associated with colon and lung cancer [6,10]. These studies, among many others, highlight the need for further investigation into whether targeting cholesterol metabolism may represent a novel therapeutic avenue for cancer treatment.

## 2. Cholesterol Synthesis and Exogenous Sources

In humans, cholesterol can be generated endogenously or derived from exogenous dietary sources. Endogenous cholesterol generation is regulated by the enzyme 3-hydroxy-3-methylglutaryl co-enzyme reductase (HMG-CoA reductase) and is predominately synthesized in the liver, where it is packaged into low-density lipoproteins (LDL). Cholesterol can also be sourced from the diet in a process that involves the packaging of the cholesterol into chylomicrons in the intestine, which are then transported into the circulation via capillaries. Cells expressing lipoprotein lipase (LPL) can degrade the chylomicrons, releasing free fatty acids, which can then be absorbed into cells. Chylomicrons, or their remnants, are then transported to the liver where they are either degraded or re-processed into VLDL particles.

**Figure 1:**
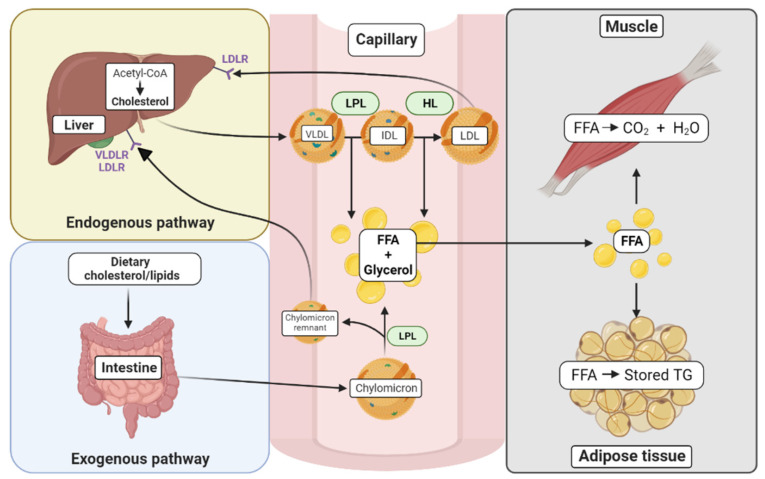
Cholesterol synthesis and trafficking in various tissues. The exogenous pathway begins with the uptake of dietary cholesterol where lipids are incorporated into chylomicrons in the intestine. Once in the circulation, the chylomicrons are metabolized by tissues that contain lipoprotein lipase (LPL), and their contents, free fatty acids (FFA) and glycerol, are metabolized by tissues such as muscle and adipose tissue. In muscle, FFAs are used for energy. In the adipose tissue, they can be stored in the form of triglycerides (TG). The resulting chylomicron remnants in the circulation are absorbed in the liver by apolipoprotein-specific receptors (VLDLR and LDLR). The endogenous pathway begins in the liver with the formation of cholesterol from acetyl-CoA, catalyzed by the enzyme HMG-CoA reductase. The VLDL particles, containing triglycerides and cholesterol esters, can be hydrolyzed by LPL to form IDL with the release of FFAs. IDLs can further be hydrolyzed to LDLs by the action of hepatic lipase (HL), which also results in the release of FFAs. LDL can be internalized by other tissues, such as the liver by the LDL receptor (LDLR).

Cholesterol homeostasis is tightly regulated by a number of processes, which are summarized in Figure 1. When dietary cholesterol levels are low, more is synthesized via the endogenous pathway. Cells that rely on endogenously synthesized cholesterol are often incapable of accumulating excess cholesterol due to homeostatic regulation at various points in the cholesterol synthesis pathway. In cancer, cholesterol homeostasis is often disordered, which may be an early event that occurs within 2 weeks of exposure to carcinogens [11]. However, it is unclear whether cholesterol increases the risk of cancer development, or whether increased cholesterol levels are a consequence of malignancy. Here, we focus on the role of cholesterol in the B-cell malignancy, chronic lymphocytic leukemia (CLL). We have recently shown significant differences in lipid metabolism in CLL cells compared to healthy B-cells [12], suggesting that cholesterol may play a crucial role in this disease.

## 3. Chronic Lymphocytic Leukemia (CLL)

CLL is the most common leukemia in the Western world. While the median age of diagnosis is 72 years, CLL is often diagnosed in younger individuals, with almost 15% of patients being 55 years or younger [13]. The clinical course of CLL is heterogenous, with many patients not requiring treatment at diagnosis. Various genetic markers, such as the presence of genomic aberrations and the mutational status of the immunoglobulin heavy chain gene (*IGHV*), are predictive of prognosis in CLL. Approximately 30% of patients will never require treatment [14], while the outcome for the remaining 70% will depend on various factors, such as comorbidities, the ability to deliver optimal treatment, and cytogenomic profiles. Historically, combined chemoimmunotherapy regimens were the preferred first-line treatments for symptomatic CLL patients (and remains so in some parts of the world due to drug accessibility). Increasingly, small molecular inhibitors, such as the BTK-inhibitor ibrutinib and the BCL-2 antagonist venetoclax, are being used as first-line treatment and at relapse. Despite high-response rates following treatment with these agents [14], most patients will ultimately relapse. Therefore, CLL is still widely considered incurable.

## 4. Serum Cholesterol Levels and Prognosis in CLL

Studies suggest that rates of dyslipidemia are higher among CLL patients than age-matched healthy controls [15]. One study showed that CLL patients suffer from hypercholesterolemia, observing elevated LDL levels in 75% of CLL patients [16]. Another study reported lower serum levels in CLL patients compared to healthy individuals, with CLL patients averaging 151.2 +/− 11.2 mg/dL compared to 195.3 +/− 8.5 mg/dL in healthy, aged-matched controls [17]. CLL patients with hypocholesterolemia have a shorter overall and treatment-free survival [18], possibly due to increased catabolism of LDL and impaired hepatic lipoprotein synthesis [17], or increased expression of the LDL receptor (LDLR) on CLL cells [19], leading to increased cellular uptake.

Hypercholesteremia may be due to an increase in the hepatic secretion of cholesterol into the blood stream; this may potentially fuel the rapid proliferation of leukemic cells in patients with a poor prognosis [20]. This notion is supported by a study that focused on patients with high-risk disease, defined by unmutated *IGHV* status (UM–*IGHV*). Using a metabolomic approach, significantly higher levels of serum cholesterol and LDL fatty acid side chains were observed in patients with high-risk disease [20]. This study also determined that UM–*IGHV* patients had higher levels of VLDL and lower levels of HDL cholesterol compared to patients with mutated *IGHV* genes (M–IGHV), while LDL levels were similar between the two groups of patients [20]. Hyperlipidemia may lead to inflammation, which helps drive CLL cell proliferation [21]. It is not surprising that statins, commonly used to treat hypercholesteremia, delayed the need for treatment by nearly 3 years [16].

In another study, no association between serum levels of LDL, HDL, total cholesterol and triglycerides, and clinical outcomes was observed [22]. However, this study included samples from a relatively small number of patients (*n* = 26), which raises the possibility that discrepancies between the studies may, at least in part, be due to the heterogeneity among CLL patients, and that much larger cohort studies are warranted. While there are conflicting reports concerning the role of cholesterol in CLL, there is clear evidence that cholesterol uptake and metabolism is disordered in CLL cells compared to healthy B-cells and other blood cancers, including acute lymphoblastic (ALL), acute myeloid (AML), and chronic myeloid leukemia (CML) [23].

## 5. The Role of Intracellular Cholesterol in CLL

Under normal physiological conditions, free cholesterol in the plasma membrane is not static and continuously shuttled between the cell interior and surface [24]. Generally, cytoplasmic cholesterol exists in the form of cholesterol esters, whereas free cholesterol is located almost entirely within the plasma membrane. The esterification of intracellular cholesterol is essential, as free cholesterol in the cytoplasm is toxic [25,26]. Intracellular free cholesterol is esterified by acyl-coenzyme-1 (ACAT-1) and released by the action of neutral cholesterol ester hydrolase 1 (nCEH-1). In normal lymphocytes, approximately 90% of intracellular cholesterol is free, and 8% is in the esterified form. However, in leukemic cells (CLL and ALL), approximately 40% is esterified, and ACAT-1 expression is increased two-fold [27]. Leukemic cells appear to have a unique ability to trap and store cholesterol in the form of lipid droplets, which may restrict the shuttling of cholesterol to cell membranes. This presents a potentially novel therapeutic avenue in CLL; by inhibiting cholesterol esterification and its incorporation into lipid droplets, increased free cholesterol levels in the cytoplasm would lead to lipotoxic cell death. This is supported by a study in which the inhibition of cholesterol esterification by progesterone or SaH 58-035 led to a reduction in cholesterol esterification and leukemic cell proliferation [27].

### Intracellular Cholesterol Levels in CLL

Almost 50 years ago, Hildebrand et al. [28] determined that CLL cells contain higher concentrations of lipids than non-leukemic cells or malignant cells from patients with CML or acute leukemia. The study determined that a significant proportion of the neutral lipid content of CLL cells is cholesterol, and that the ratio of free-to-total cholesterol is elevated in CLL cells compared to healthy B-cells. CLL cells were also determined to contain high levels of phospholipids. However, another study, published around the same time as the Hildebrand study, contradicted these findings by demonstrating that the cholesterol content of CLL cells was significantly lower than that of healthy B-cells [29]. Lower intracellular levels of cholesterol have also been observed in CLL cells than in leukemic cells from patients with hairy cell leukemia [30]. A recent study also concluded that there was no difference in total intracellular cholesterol or phospholipid levels in CLL cells compared to healthy B-cells, but that the leukemic cells have lower levels of free cholesterol and higher levels of cholesterol esters than their healthy counterpart [27]. Reasons for the discrepancies between these studies are unclear but could be due to the proliferative state of the CLL cells examined. CLL cells migrate from tissue microenvironments in lymph nodes and bone marrow to the peripheral blood, with concomitant changes in their metabolic demands; quiescent CLL cells in the peripheral circulation likely have lower metabolic demands, which may be reflected in their cholesterol content.

## 6. Cholesterol Uptake in CLL

### 6.1. HDL Uptake

**Figure 2:**
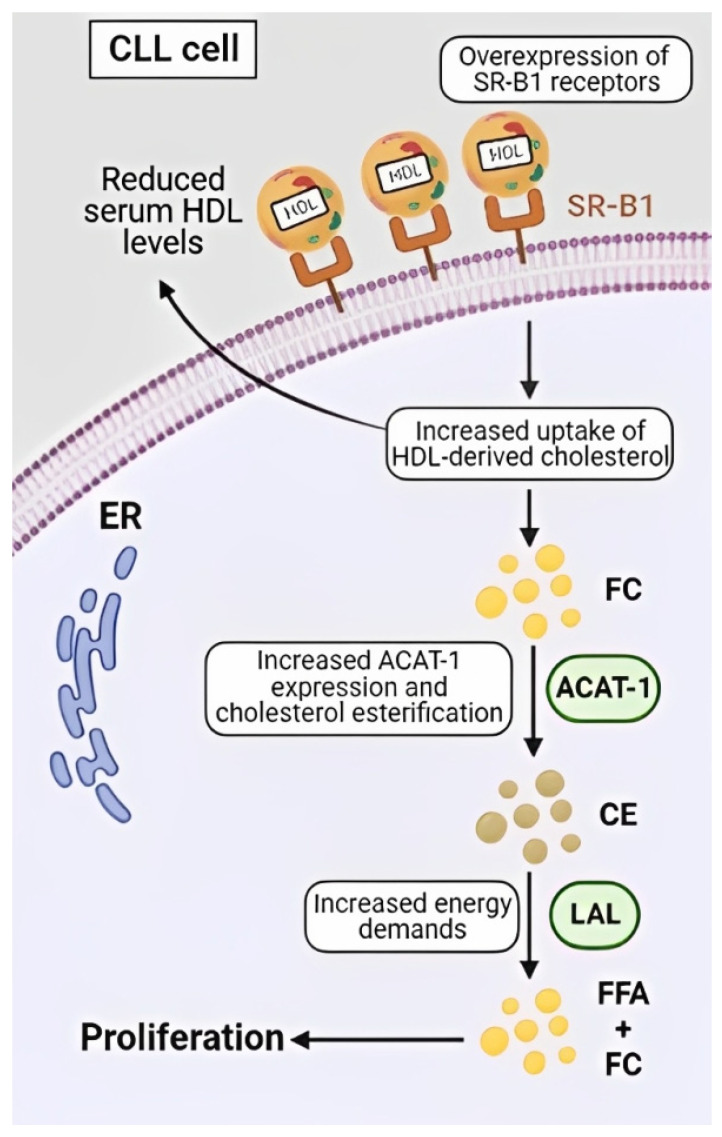
The high-affinity HDL receptor, SR-B1, is overexpressed on CLL cells, which leads to an increased uptake of HDL-derived cholesterol esters and the diffusion of free cholesterol (FC) into the plasma membrane and cytoplasm. As cytoplasmic FC is toxic, it undergoes esterification by acyl-coenzyme-1 (ACAT-1) to protect the cells from ER stress and apoptosis. Cholesterol esters (CE) represent a safe way to store intracellular cholesterol and act as a repository, which can be exploited by the cancers cells to fuel rapid proliferation by lysosomal acid lipase (LAL) degradation of CE to free fatty acids (FFA) and FC.

Many malignant cells overexpress SR-B1, a high affinity receptor located in lipid rafts that functions as an HDL receptor and mediates cellular uptake of HDL-derived cholesterol esters [31,32]. When HDL particles bind to SR-B1, the diffusion of free cholesterol occurs from the HDL particle into the plasma membrane, resulting in a reduction in the size of the HDL particle. Decreased levels of HDL have been observed in the peripheral blood of CLL patients compared to aged matched healthy controls [33], which may be due to the overexpression of SR-B1 on CLL cells [34], leading to an increase in the sequestration of HDL into tumour cells (Figure 2).

In B-cell lymphomas, the administration of synthetic HDL particles, which mimic endogenous HDL particles but are devoid of any free cholesterol or cholesterol esters, have been proposed as a therapeutic option [35]. These HDL nanoparticles were found to potently induce apoptosis of the leukemic cells in animal models without any evidence of systemic side effects or toxicity [35]. A similar study in CLL [34] determined that expression of SR-B1 on CLL cells, but not healthy B-cells, correlated with the cytotoxic effects of the nanoparticles towards the leukemic cells, but not their healthy counterpart. Although HDL nanoparticles are yet to be tested in human trials, the preclinical data suggests they may represent a novel therapeutic approach for CLL and other lipid-avid cancers.

### 6.2. LDL Uptake

**Figure 3:**
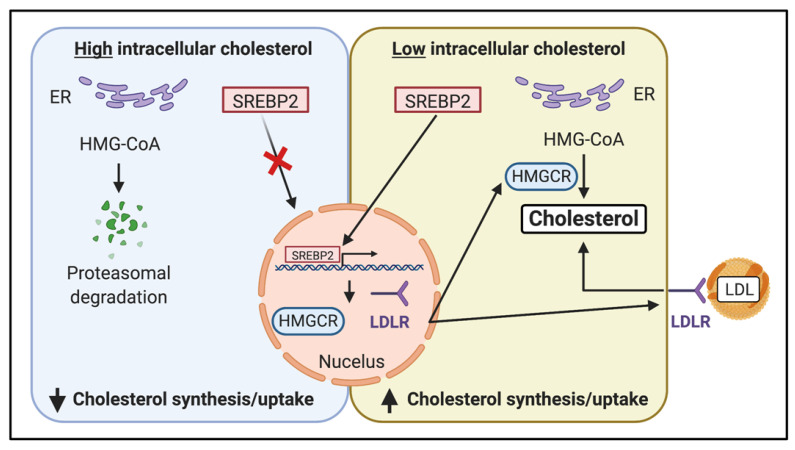
The sterol regulatory element-binding protein transcription factor 2 (SREBP2) pathway regulates endogenous cholesterol synthesis. Cholesterol levels in the endoplasmic reticulum (ER) act as a sensor for intracellular cholesterol homeostasis. When high-intracellular cholesterol levels are detected, SREBP2 remains in the ER, resulting in a decreased expression of HMG–CoA reductase (HMGCR). HMG–CoA also undergoes proteasomal degradation, further reducing its activity. When cholesterol levels in the ER decrease, SREBP2 translocates to the nucleus and activates the transcription of genes, including *HMGCR,* which regulates the expression of HMG–CoA reductase and the LDL receptor (LDLR), resulting in an increase in cholesterol uptake into the cell.

The internalization of exogenous cholesterol is predominately mediated by LDL receptors (LDLR) and endocytosis [36]. When LDL particles come in contact with cancer cells, they bind to specific LDLRs. The LDL particle and its associated receptor are then internalized by clathrin-mediated endocytosis. The LDL and LDLR are then transported to lysosomes and hydrolyzed to release cholesterol and fatty acids, which are then incorporated into various cellular structures, including membranes [37]. LDLR expression is regulated by sterols via sterol regulatory elements (SRE), which bind to transcription factors known as SRE-binding proteins (SREBPs). In response to elevated levels of intracellular cholesterol, the expression of LDLRs and SREBP2 is downregulated [38]. In healthy cells, SREBP2 is a potent regulator of the *LDLR* promoter, but this does not appear to be true in cancer cells [39] (Figure 3).

In CLL, LDLRs may play a critical role in regulating CLL proliferation. Sankanagoudar et al. [24] observed hypocholesteremia and hyper-expression of LDLR in CLL cells, which was associated with increased levels of cholesterol in both the nucleus and cytoplasm. The study demonstrated that the uptake of fluorescently conjugated cholesterol (Dil–LDL) was significantly higher in CLL lymphocytes than in healthy B-cells [19]. However, the study also determined that the rate of LDL degradation is lower in CLL cells than in B-cells from healthy individuals, and that LDLR activity was no different in CLL cells than in healthy B-cells [40]. Similar results were observed by Juliussion and Vitols, in which the authors used radio-labelled LDL (^125^I-LDL) to demonstrate that the rate of LDL degradation is significantly lower in CLL cells compared to healthy B-cells [40]. LDLR activity has also been shown to be lower in CLL cells than in leukemic cells from patients with acute myeloid leukemia (AML) [41]. The low LDLR activity in CLL cells observed in these studies may be due to the quiescent nature of CLL cells in the peripheral circulation, which are morphologically similar to small resting B-cells [42,43]. A similar observation was noted in quiescent fibroblasts. When these cells were stimulated with growth factors, there was an increased expression of the LDLR at the cell surface [44] However, no studies to date have compared LDLR activity in CLL cells to that of small resting lymphocytes, probably due to the rarity of the latter in healthy individuals. As the authors suggest, the variability in LDL activity observed may also be due to heterogeneity between the CLL patients studied [40]. Although further studies are required to determine whether LDL uptake correlates with clinical parameters in CLL, it is conceivable that LDL utilization may promote leukemic cell proliferation and may, therefore, be associated with a more aggressive disease course.

### 6.3. Chylomicron Metabolism

Chylomicrons are lipoproteins that transport lipids in the blood. Chylomicrons are degraded through lipolysis by the enzyme lipoprotein lipase (LPL) at the cell surface. LPL hydrolyses phospholipids in VLDL particles, resulting in an increase in serum triglycerides, a release of free fatty acids, and the conversion of VLDL particles to LDL particles. Remnants of chylomicrons are then either degraded or re-processed into VLDL particles by the liver. LPL is highly expressed on the surface of CLL cells [45,46] but not healthy lymphocytes, suggesting the leukemic cells are capable of metabolizing chylomicrons. Furthermore, high LPL expression on CLL cells is associated with a poor prognosis [47] and significantly worse survival [48]. Data from our laboratory have shown that CLL cells include high amounts of free fatty acids [49], which supports the hypothesis that free fatty acids may provide a crucial energy source for these cells.

Chylomicron degradation is very difficult to assess in vivo. Sakashita et al. [50] created a double-labelled chylomicron-resembling emulsion, which was injected into 10 CLL patients and 11 healthy controls. The kinetics of triglycerides (TAGS) and cholesterol ester levels were then determined in plasma samples collected over a 30-min time course. No significant difference in the fractional clearance rate of TAGS and cholesterol esters was observed between CLL cells and controls, suggesting that the rates of chylomicron lipolysis and remnant removal are not altered in CLL. However, ~90% of the patients in this study had indolent disease suggesting that the energy requirements and proliferation rates of the leukemic cells may be lower than in patients with rapidly progressing disease.

## 7. LDLs as Signaling Molecules

Numerous pathways mediate the growth and proliferation of CLL cells, but three appear to be critical: the extracellular signal-regulated kinase, mitogen-activated protein kinase (ERK1/2-MAPK), and Ak-strain transforming (AKT) and signal transducer and activator of transcription-3 (STAT3) pathways, which are all activated by LDLs in CLL cells [23]. STAT3 phosphorylation drives LPL expression by binding to transcriptional activators [51]. The breakdown of VLDL and chylomicrons by LPL, and the resulting free fatty acids, activate STAT3 and, in turn, increase the expression of LPL [52]. However, they do so without cross-linking of the B-cell receptor (BCR) [23]. LDL uptake has been observed in healthy PBMCs, although no change in plasma cholesterol levels or activation of STAT3 was observed [23], reinforcing the notion that CLL cells handle cholesterol differently than normal B-cells. Interestingly, the downregulation of several genes involved in intracellular cholesterol transport has been observed in CLL cells compared to healthy B-cells. These include sortilin-related receptor-1 (SORL1), which inhibits lipolysis of intracellular lipid droplets. CLL cells also appear to handle cholesterol differently than tumour cells from other blood cancers, including the Burkitts lymphoma cell line Daudi, which showed no increase in STAT3 phosphorylation in response to the addition of exogenous LDLs. STAT3 phosphorylation in CLL cells in response to LDLs can be suppressed by anti-IL-10 antibodies and by the inhibition of Janus kinase (JAK) [53], suggesting that there is some overlap in the function of these pathways. In their study, McCaw et al. [23] investigated which constituents of LDL molecules were responsible for activation of STAT3 and determined that long chain fatty acids and free cholesterol were critical, and that short- and medium-chain fatty acids, which are not transported in LDLs, did not activate STAT3 [23]. This is supported by our recent study, in which we have shown that CLL cells preferentially include long chain fatty acids rather than medium- and short-chain fatty acids [49].

## 8. Endogenous Sterol Synthesis Pathways

CLL cells respond to extracellular LDL by reducing cholesterol synthesis [54]. However, it has been suggested that CLL cells may be more dependent on cholesterol synthesized by the endogenous pathway than on the LDLR-mediated uptake [55]. Sterol synthesis is tightly regulated by the enzyme HMG–CoA reductase and cholesterol levels in the endoplasmic reticulum (ER) act as a sensor for intracellular cholesterol homeostasis. When levels of cholesterol at the ER decrease, sterol regulatory element-binding protein transcription factor 2 (SREBF2) translocates to the nucleus and activates transcription of genes, including HMG–CoA reductase (*HMGCR*), which induce cholesterol synthesis and increase LDLR expression, resulting in increased uptake of cholesterol into the cell. When intracellular levels of cholesterol rise, the expression of HMG–CoA reductase is decreased through proteasomal degradation and a reduction in gene transcription (Figure 3).

HMG–CoA reductase activity is significantly elevated (up to 20-fold) in a wide variety of hematological malignancies, including in CLL cells, (35.1 ± 5 pmol/min/mg) compared to healthy B-cells (10.3 ± 0.8 pmol/min/mg) [55]. HMG–CoA reductase activity has been demonstrated to be regulated by the fluidity of the microsomal membrane, which are small vesicle-like artifacts formed from the ER. Increased membrane fluidity has been described as a feature of CLL lymphocytes [56,57], possibly due to low membrane cholesterol levels and hypolipidemia observed in several studies [58,59,60]. It is possible that the increase in membrane fluidity in CLL cells drives the increase in HMG–CoA reductase activity in these cells [61]. Several reports suggest that the basal phosphorylation state of the enzyme may be altered in cancer cells [62,63] leading to an increase in activity of the enzyme. Since HMG–CoA reductase expression is higher in CLL cells than in their healthy counterpart [55], it is likely that CLL cells may be more sensitive to HMG–CoA reductase inhibitors, such as statins, which will be discussed in more detail later in this review. High levels of endogenous sterol synthesis are likely to be crucial, particularly during periods of rapid cell proliferation, as high amounts of cholesterol are essential for the generation of new cellular structures, including plasma membranes. Newly synthesized cholesterol produced via the endogenous pathway can be incorporated into the plasma membrane in less than 15 min after synthesis [64] facilitating this rapid cell proliferation.

## 9. The Use of Cholesterol Lowering Drugs in CLL

Numerous cell types obtain cholesterol from LDLs, which circulate in the blood. Statins, a widely used class of cholesterol-lowering drugs, decrease LDL levels by inhibiting HMG–CoA reductase [65]. This, in turn, increases LDL clearance from the circulation by increasing expression of the LDL receptor on the cell surface. While the benefits of statins in cardiovascular disease are well-documented, they have also been associated with a reduction in cancer-associated deaths [66,67]. Multiple cancer-related studies have been published in which the effects of statins, and a range of other lipid-lowering medications, have been examined both in vitro (summarized in Table 1) and in clinical studies (summarized in Table 2).

### 9.1. Statins

**Figure 4:**
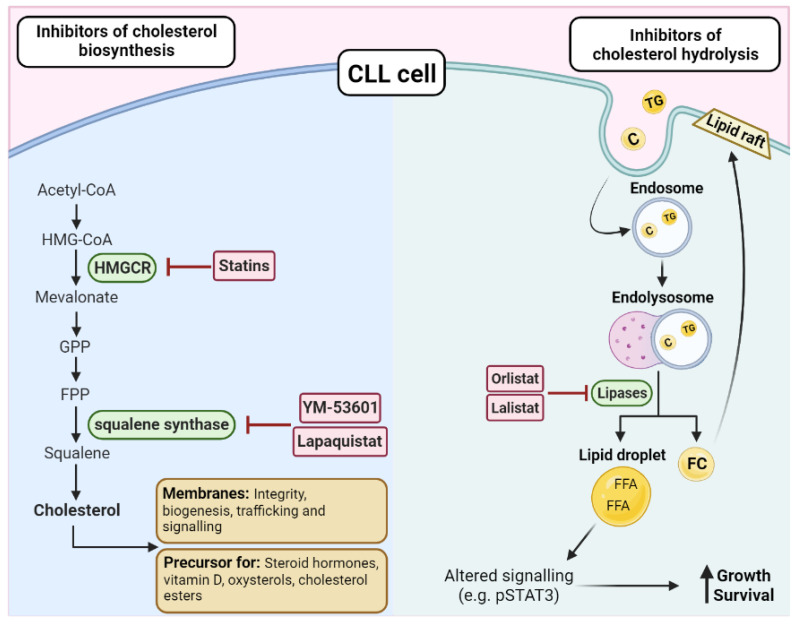
Targeting cholesterol biosynthesis or hydrolysis in CLL. There are two key enzymes in the biosynthesis of cholesterol, HMG–CoA reductase (HMGCR), and squalene synthase, which can be targeted by a range of inhibitors such as statins, YM-53601, and lapaquistat. As cholesterol is essential for membranes and as a precursor for cholesterol esters and other factors, disrupting the biosynthesis pathway is an attractive treatment option in CLL. On the other hand, triglycerides (TG) and cholesterol (C) can directly enter the cell via endosomes and be hydrolyzed by lipases. The resulting free fatty acids (FFAs) and free cholesterol (FC) can have downstream effects that promote the growth and survival of the CLL cells, such as altering the expression of transcriptional regulators, such as STAT3 or by increasing the lipid content of the membrane, leading to altered cellular functions, such as increased activation of oncogenic pathways and trafficking. Orlistat and lalistat are two inhibitors that have been investigated in CLL due to their ability to block lipases and prevent the hydrolysis of cholesterol and triacylglycerols.

Statins are a broad class of drugs that include hydrophilic (rosuvastatin and pravastatin) and lipophilic statins (atorvastatin, simvastatin, lovastatin, fluvastatin, cerivastatin, and pitavastatin). Hydrophilic statins are liver-specific, whereas lipophilic statins are widely distributed in different tissues. Their mechanism of action and systemic effects are summarized in Figure 4.

As HMG–CoA reductase levels are elevated in CLL cells [55], it is likely the leukemic cells are particularly sensitive to inhibitors of the enzyme, including statins. In general, CLL patients who are prescribed statins tend to be older and male [16]. Compared to patients who were not taking statins, CLL patients given these drugs were determined to have significantly lower LDL levels, lower amounts of intracellular cholesterol, fewer circulating CLL cells, and longer lymphocyte doubling times [23].

There is strong in-vitro evidence that statins also have direct tumoricidal effects against CLL cells, inducing apoptosis-mediated cytotoxicity [68,69]. Although the mechanisms of action of statins against CLL cells still have not been fully elucidated, statins have been shown to inhibit signaling via the NF-κB transcription factor in myeloid cells [81]. NF-kB signaling also plays a critical role in promoting CLL cell viability [82], and an in-vitro study determined that the statin, simvastatin, induces apoptosis of CLL cells by lowering the BCL-2/Bax ratio and through activation of caspase 9 [69]. Importantly, these pro-apoptotic effects were induced specifically in the leukemic cells and not in normal lymphocytes. There is also evidence from a study involving the short-term ex-vivo culture of primary CLL cells that simvastatin may act in synergy with the purine analogues, fludarabine, and cladribine [71]. Furthermore, simvastatin was found to be equally effective at inducing apoptosis in cells from high-risk CLL patients and patients with a more favorable prognosis [71].

To date, there have been seven clinical studies (summarized in Table 2) that have investigated the effects of statins on the pathogenesis of CLL [15,16,22,77,78,79,80]. Of these studies, five concluded that statins have favorable effects on CLL patient outcomes [15,16,78,79,80] by either reducing disease progression [16], reducing the risk of CLL development [80] or by increasing overall survival [15,78,79]. The outcome data in these studies were derived from a large number of patients (> 4000), with varying ages and included treatment naïve patients and patients with relapsed/refractory disease. The other two studies [22,77] observed no impact of statins on clinical outcomes. One of these studies, in which statins were administered to newly diagnosed Rai Stage 0 patients [77], determined that statins had no effect on time to initial therapy. The study also included patients that were already taking statins at the time of their CLL diagnosis but concluded that statins had no impact on the time to first treatment in this group of patients. Similarly, Friedman et al. observed no effect of statins on treatment free survival but did determine that patients who were taking statins at the time of CLL diagnosis were less likely to require immediate therapy. However, as the authors pointed out, the retrospective analysis and short follow-up time may be confounding factors that limit the power of the study to identify differences between the patient groups.

### 9.2. Other Cholesterol Lowering Drugs in CLL

The lipase inhibitors, lalistat and orlistat, also function through effects on cholesterol homeostasis. Lalistat is a small molecular inhibitor that inhibits lysosomal lipases (Figure 4). Following cellular uptake, LDL particles are degraded in lysosomes, releasing cholesterol and free fatty acids. In CLL cells, cholesterol esters are further degraded by lysosomal lipases, which generate free fatty acids and increase the activity of the pro-survival STAT3-mediated signaling pathway. Lalistat inhibits this process by preventing degradation of cholesterol esters, which leads to a reduction in the phosphorylation and activity of STAT3 [23]. Orlistat, marketed under the name xenical, is a lipase inhibitor that prevents adsorption of dietary fats. Orlistat inhibits lipoprotein lipase (LPL) and, therefore, prevents the release of free fatty acids from LDL particles. In the only published study to date, orlistat was observed to induce apoptosis of primary CLL cells in vitro [70].

Statins have been demonstrated to reduce surface expression and induce conformational changes of the CD20 molecule on B-cells and decrease the cytotoxic effects of the anti-CD20 monoclonal antibody, rituximab, towards CLL cells [83]. The possibility that statins may reduce the clinical efficacy of CD20-targeted therapies [84] is an important consideration as chemoimmunotherapeutic regimens that include anti-CD20 antibodies are still commonly used to treat CLL. An alternative approach may be to use agents that target other critical regulators of cholesterol biosynthesis, particularly for patients who are concurrently receiving anti-CD20 therapies. A study of two inhibitors of squalene synthase (YM-53601 and TAK-475) and an inhibitor of 2-3,oxidosqualene cyclase (BIBB-515) determined that these compounds reduce intracellular cholesterol levels by around 20% and increase CD20 surface expression in the MEC2 cell line [73]. Consistent with the increase in CD20 expression, both inhibitors were found to significantly increase the sensitivity of the cell line and primary CLL cells to rituximab and, also the purine analogue, fludarabine. These data suggest that certain drugs that target regulators of cholesterol biosynthesis may have both direct cytotoxic effects and increase the sensitivity of CLL cells to current therapies, including regimens containing anti-CD20 therapies.

## 10. Conclusions

Cholesterol is well recognized as an essential nutrient for cells throughout the body. It is an important precursor in the production of hormones, bile acids, and Vitamin D, and it is a critical structural component of cell membranes. The malignant transformation of healthy B-cells to CLL cells is associated with the reorganization of membrane components, which is dependent on cholesterol. CLL cells appear capable of overriding normal feedback mechanisms that regulate cholesterol levels, resulting in elevated intracellular levels of the lipid, a greater intracellular mass, modified membrane composition, and alterations in signaling pathways. However, there are conflicting reports in the literature concerning the role of dietary cholesterol in the pathobiology of CLL.

Metabolic syndrome is a well-known risk factor for the development and prognosis of cancer, and one important aspect of metabolic syndrome is dyslipidemia. Although further research is required to determine the causes of cholesterol dysregulation or dyslipidemia in cancer, targeting pathways involved in cholesterol metabolism has been proposed as a unique therapeutic approach in many cancers, including glioblastoma [85] and cancers of the breast [86] and prostate [87]. There is also evidence that cholesterol-lowering drugs, such as statins, may reduce the risk of tumorigenesis. Several studies have examined the potential impact of statins on the outcome of patients with CLL, but short follow-up times and retrospective analyses limit interpretation of the data, particularly concerning serum triglyceride and cholesterol levels, which must be assessed under fasting conditions. However, the findings of these studies raise some potentially important points, including the possibility that concurrent administration of statins may significantly impact the response of patients to regimens containing anti-CD20 antibodies [84].

While the tumoricidal effects of drugs that inhibit both endogenous cholesterol synthesis and inhibitors of HMG–CoA reductase highlight the critical role of cholesterol in CLL cell survival and proliferation, there are still many unanswered questions. For example, does cholesterol play a role in drug resistance in CLL or in the transformation of the disease to a more aggressive B-cell malignancy, known as Richter’s transformation? In other cancers, cholesterol is known to play a role in drug-resistance [reviewed in [88]]. Therefore, it is conceivable that agents that target cholesterol metabolism may restrict the development of drug resistance and transformation in CLL.

Microenvironments within lymph nodes and bone marrow promote survival and drive the proliferation of CLL cells. Cholesterol may play an important role in supporting the high energy demands associated with the rapid CLL cell proliferation that occurs in these microenvironments. However, no studies to date have examined whether lipids and lipoproteins represent a crucial energy source for CLL cell proliferation and play a role in mediating the interaction of CLL cells with accessory cells within the tumour microenvironment.

Further studies concerning the potentially significant role of dietary cholesterol in CLL are also required, particularly as dietary intervention studies are relatively easy to conduct, with little risk to the patients. As many CLL patients are often initially managed with “watch-and-wait” approaches, it remains to be explored whether dietary changes may delay the need for treatment, particularly in patients with low- or medium-risk disease. Addressing these critical questions and gaining a better understanding of the role of cholesterol in the pathogenesis of CLL may identify novel treatment or management strategies.

## Figures and Tables

**Table 1:** Outcomes from in-vitro and animal studies on the effects of targeting cholesterol-associated pathways in CLL.

Year	Compound(s)	Model(s)	Study Summary	Dependent Variables Assessed	Key Findings	Reference
1984	Mevastatin	Primary CLL cells	Assessed cholesterol content in the plasma membrane of CLL cells with and without mevastatin (compactin) treatment	Filipin–cholesterol complexes within the plasma membrane and cell growth	Mevastatin reduced plasma membrane cholesterol in CLL cells, even in the presence of lipid-rich serum	[54]
1997	Simvastatin, lovastatin, pravastatin	Primary CLL cells	Investigated multiple statins as inhibitors of CLL cell proliferation	Mitogen-induced thymidine uptake in presence and absence of statins	Statins inhibited thymidine uptake in a concentration-dependent manner	[68]
2003	Simvastatin	Primary CLL cells	Assessed response of primary CLL cells to simvastatin	Viability-, apoptosis-, necrosis-, apoptotic-signalling cascade	Significant reduction in CLL cell viability and a significant increase in apoptosis and necrosis	[69]
2008	Orlistat	Primary B-cells from CLL patients and healthy control donors	Examined the inhibitory effects of orlistat on primary CLL and healthy B cells	Apoptosis, viability, and lipase activity	Orlistat induced apoptosis in primary CLL cells and acted in synergy with fludarabine. Effects were reduced by BCR stimulation.	[70]
2010	Simvastatin	Primary peripheral blood and bone marrow mononuclear cells isolated from newly diagnosed, untreated CLL patients	Ex-vivo assessment of simvastatin alone and in combination with fludarabine and cladribine	Apoptosis and DNA damage	Simvastatin induced tumour-specific apoptosis when administered alone and with the purine analogues	[71]
2013	Atorvastatin	Primary CLL cells	Evaluated the effect of atorvastatin on primary CLL cells	Apoptosis, viability, expression of proteins that regulate apoptosis	Atorvastatin induced apoptosis in CLL cells, but not healthy mononuclear cells	[72]
2013	Atorvastatin	Primary CLL cells	Investigated the effect of atorvastatin on lymphocytes from CLL patient cells	Apoptosis and expression of CD5, CD38, ZAP-70, and Annexin V	Atorvastatin increased apoptosis and decreased expression of CD5 and ZAP-70 in patient CLL cells	[33]
2014	BIBB-515, YM-53601 and TAK-475 (cholesterol lowering agents)	MEC-2 CLL cell line and primary CLL cells	Explored effects of agents that target cholesterol synthesis on the efficacy of anti-CD20 chemoimmunotherapeutic regimens	Total cholesterol levels, CD20 expression, and cell viability	Agents reduced total cholesterol levels, increased CD20 expression and chemoimmuno-sensitivity to various therapeutic agents	[73]
2015	Simvastatin	Primary CLL cells	Comparison of *IGHV* mutated and unmutated multidrug resistant CLL cells to identify therapeutic targets	Cholesterol levels, activation of pathways (Ras/ERK1-2, RhoA/RhoA, HIF-1α/P-glycoprotein axis)	Increased activation of Ras/ERK1–2 and RhoA/RhoA kinase-signalling pathways conferred drug resistance in CLL cells, which was countered by simvastatin	[74]
2017	Lalistat	Primary CLL cells	Functional analysis of the role of low-density lipoproteins (LDLs) in CLL	CLL cell count, and assessment of lipid droplet and membrane cholesterol content, phosphorylated STAT3 and cytokine levels	LDL adminstration increased levels of cholesterol in plasma membrane, increased phosphorylation of STAT3 and CLL cell number, indicating a proliferative effect	[23]
2018	Simvastatin	Primary CLL cells, AML and DLBCL cell lines, C57BL/6N mice injected with lymphoma cells	Assessed effects of simvastatin, with and without venetoclax, on cell lines and primary cells from a variety of lymphomas and leukemias	Viability, protein geranylgeranylation, levels of pro-apoptotic PUMA protein	Statin-mediated inhibition of HMGCR enhanced antitumor effects of venetoclax (decreased protein geranylgeranylation and increased expression of PUMA)	[75]
2020	Simvastatin, lovastatin, fluvastatin, rosuvastatin	HG3 and MEC-1 CLL cells lines, Primary CLL cells	An in-silico and in-vitro approach to identify drug and drug combinations for targeting CLL cells in the tumour microenvironment	CLL cell viability, cytotoxicity, and proliferation	Simvastatin potentiated the cytotoxic effects of venetoclax and ibrutinib	[76]

PUMA = p53 upregulated modulator of apoptosis (proapoptotic protein); DLBCL = diffuse large B-cell lymphoma.

**Table 2:** Clinical studies assessing the use of cholesterol lowering drugs on various clinical outcomes in CLL patients.

Year	Study Summary	Subjects (n)	Results	Key Findings	Reference
2010	Evaluated the effects of statins on clinical outcomes and rituximab efficacy	Newly diagnosed Rai stage 0 CLL patients (n = 686)	TFT and OS were not significantly different between CLL patients who were or were not taking statins at time of diagnosis (TFT 7.9 with statins vs. 11.8 years without, *p* = 0.52; OS 10.1 with statins vs. 11.4 years without, *p* = 0.1)	Statins did not appear to impact the clinical outcomes assessed or rituximab efficacy	[77]
2010	Assessed overall survival (OS) and treatment free survival (TFS)	CLL patients taking statins at diagnosis (n = 254)	No significant difference in TFT or OS between CLL patients who were or were not taking statins at diagnosis	Administration of statins at time of diagnosis did not affect the clinical outcomes assessed	[22]
2014	Explored whether statin use could be a predictor of survival in CLL patients	CLL patients (n = 130)	Statin use was able to predict patient overall survival in a multivariate analysis (*p* < 0.017)	Statin use was a predictor of survival in older patients (median age = 72) with early-stage disease and an ECOG performance status of 1–2.	[78]
2014	Retrospective analysis of CLL patients treated with or without adjunct statin and/or aspirin treatment	Relapsed/refractory (R/R) CLL patients treated with FCR as a salvage therapy (n = 280)	Patients receiving both statins and aspirin (PFS—6.1 years, OS—9.2 years) vs. PFS—1.6 years, OS—3.7 years in patients not receiving statins (PFS *p* = 0.003; OS *p* = 0.05). CLL patients receiving both statins and aspirin had a 66% reduced risk of disease progression and a 60% reduced risk of death (PFS hazard ratio [HR] = 0.34, 95% confidence interval [CI] = 0.18–0.65, *p* < 0.001; OS HR = 0.40, 95% CI = 0.21–0.79, *p* = 0.008).	Administration of statins and aspirin was associated with a significant improvement in response rate and survival in R/R CLL patients treated with FCR as a salvage therapy	[79]
2016	Reviewed clinical data to establish a link between hypercholesterolaemia and CLL	CLL patients (n = 231)	Time to first treatment (TFT) was longer in CLL patients receiving statins (57.5 (IQR = 32, 77) vs. 36 (IQR = 11, 100) months, *p* < 0.02)	Study suggests a high incidence of hypercholesterolaemia in CLL patients, and that statins may reduce disease progression	[16]
2016	Population-based, case-control study investigating a potential association of dyslipidaemia with CLL	CLL patients > 66 years of age (n = 2124) and matched healthy controls (n = 7935)	Statin use prior to or after CLL diagnosis was associated with an improvement in OS (7.8 years, 95% CI 7.3 to 8.4 vs. 4.1 years, 95% CI 3.7 to 4.5 years, *p* < 0.001)	Statins and other lipid-lowering medications was associated with improved survival rates in CLL patients	[15]
2019	Nested case-control study examining the risk of CLL among statin users. Patients were grouped by chemical and pharmacodynamic properties of the medication	CLL patients > 40 years of age (n = 1385) and healthy controls matched based on gender, age and country of residence (n = 6841)	Low-potency lipophilic statins were associated with a lower risk of CLL (OR = 0.64, 95% CI 0.45–0.92), particularly in patients who more regularly received the stains (OR = 0.44, 95% CI 0.22–0.88). Administration of hydrophilic or high-potency lipophilic statins had no effect on incidence of CLL.	Administration of low-potency lipophilic statins was associated with a dose-dependent reduction in risk of CLL development	[80]
